# Progestins - a review of clinical application in gynecology

**DOI:** 10.1080/07853890.2025.2570794

**Published:** 2025-12-12

**Authors:** Dominika Żyła, Dominika Kuźmiuk, Martyna Rynkowska, Małgorzata Satora, Krzysztof Kułak, Rafał Tarkowski

**Affiliations:** ^a^ Department of Oncological Gynecology and Gynecology, Students’ Scientific Association, Medical University of Lublin, Lublin, Poland; ^b^ Department of Oncological Gynecology and Gynecology, Medical University of Lublin, Lublin, Poland

**Keywords:** Progestogens, progestins, clinical application, progestins treatment, gynecology

## Abstract

**Aims:**

Progestins are a group of substances with a wide range of applications. They are classified into pregnanes, estranes, and gonanes, and further divided into four generations based on their testosterone (TE) or progesterone (P4) origin. This study reviews the existing literature on the clinical use of progestins.

**Methods:**

A literature review was conducted by PubMed, Google Scholar, Scopus and ClinicalTrials. The following search terms and their combinations were used when reviewing the aforementioned databases: (progestogen OR progestogens OR progestin OR progestins) AND (women OR woman OR men OR man OR adult). The analyzed publications about progestins were dated from 2005 to 2024.

**Results:**

Progestins are used in both women and men, either alone (progestin-only contraception, POC) or combined with other substances (combined hormonal contraception, CHC). They are primarily used for contraception but also offer therapeutic benefits for conditions like abnormal uterine bleeding, endometriosis, secondary amenorrhea, infertility, and endometrial hyperplasia. In women with a uterus, balancing progestin with estrogen is crucial to prevent endometrial cancer. Progestins are also used in menopausal hormone therapy (MHT) to alleviate menopausal symptoms and can help prevent premature deliveries. Ongoing clinical trials are exploring their broader potential.

**Conclusions:**

Progestins have diverse therapeutic uses, with promising applications in various diseases. However, off-label use may present legal risks for healthcare providers, highlighting the need for clearer guidelines, particularly in non-gynecological treatments.

## KEY MESSAGES

Progestogens, including both natural and synthetic progesterone compounds, have diverse therapeutic applications, including hormonal contraception, menopausal hormone therapy, and treatment for conditions like endometriosis and secondary amenorrhea.

The classification of progestins into different generations and types, based on their chemical structure, influences their pharmacological profiles and clinical effects, such as the potential for androgenic side effects or lack thereof in newer generations.

Ongoing clinical trials and research into progestogens’ broader therapeutic uses, including for non-gynecological conditions, offer promising prospects, but their off-label use may create legal risks, emphasizing the need for clear guidelines.

## 1. Introduction

**Figure 1.**
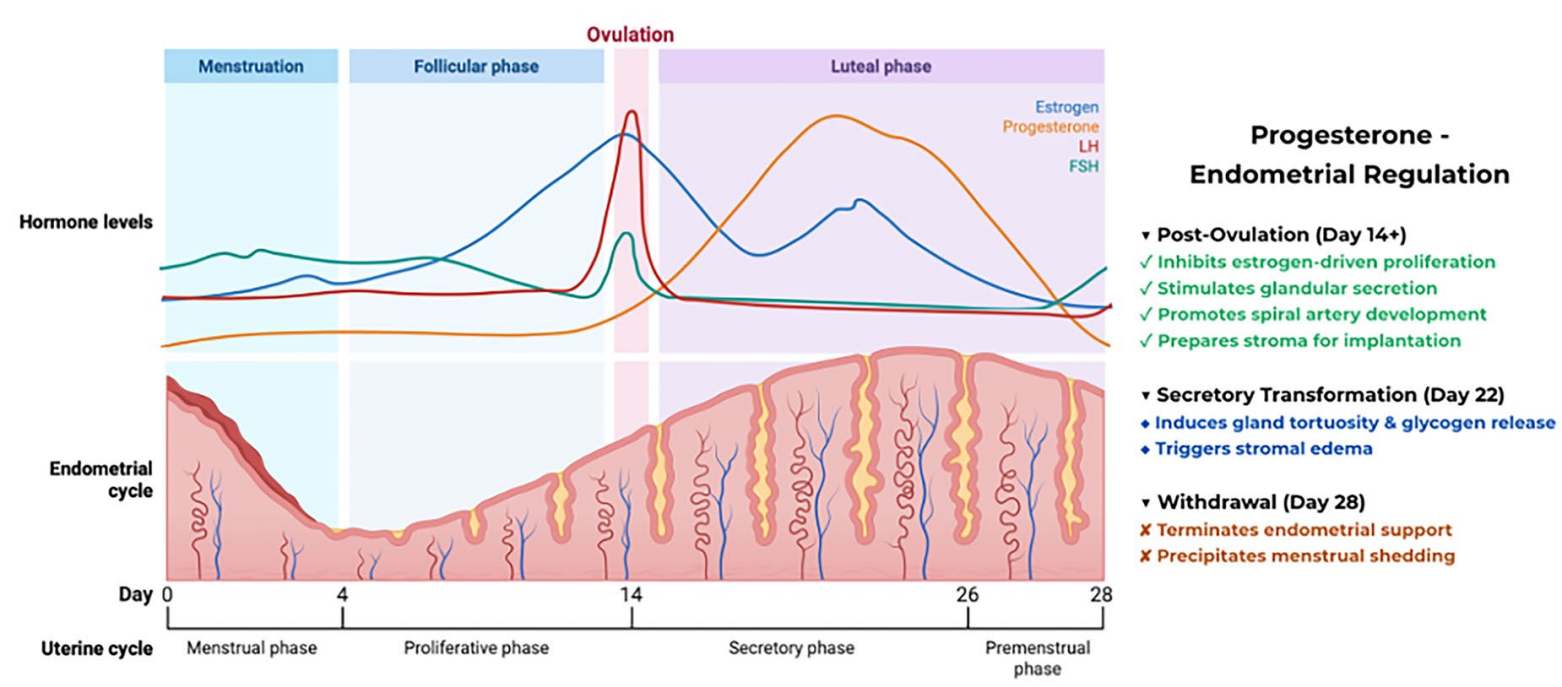
Progesterone-mediated endometrial transformation during the menstrual cycle.

Among the array of therapeutic interventions, the utilization of progesterone compounds is a fundamental aspect, harnessing the physiological actions of progesterone naturally produced within the human body [1]. Within this realm, progestogens, also referred to as gestagens or gestogens in the literature, emerge as pivotal agents with diverse applications. These include primarily the most commonly known ones, i.e. hormonal contraception in the form of progestin-only contraception (POC) or combination with other substances such as combined hormonal contraception (CHC) [1–8]. The POC includes progestin-only pills (POP), intrauterine devices with levonorgestrel (LNG-IUD) and etonogestrel implants [9]. CHC consists of combined oral contraception (COC) and non-oral combinations [1]. Interestingly, some of the mentioned methods can be applied not exclusively in women but also in men [1,10]. However, it brings benefits not only due to the avoidance of undesirable fertilization but also due to the additional benefits of using POC or COC in women struggling with other conditions, such as abnormal uterine bleeding [11]. In addition to the contraceptive effect, it is also possible to use progestins as menopausal hormone therapy (MHT) in women [12,13]. In this case, progestins are used in addition to estrogens to counterbalance their results. Estrogens increase the thickness of the endometrium and thus contribute to the increased risk of endometrial cancer (EC). Progestogens counteract this process by decreasing the number of estrogen receptors in target tissues. Moreover, they reduce the level of luteinizing hormone (LH), which is responsible for stimulating the ovaries to produce estrogens [14]. Figure 1 illustrates the role of P4 in the endometrium and the hormonal changes during different parts of the menstrual cycle. Other known applications include therapy in endometriosis, secondary amenorrhea, infertility, endometrial hyperplasia (EH), and the prevention of premature deliveries [11,15,16]. The use of progestins in cancer therapy remains controversial [11,17–20]. The studies conducted on this subject are described in our work, as well as other noteworthy works using progestogens, which constitute the prospect of new treatment options.

**Figure 2.**
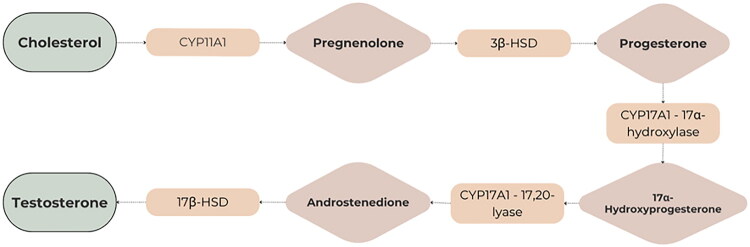
Biosynthesis of testosterone from cholesterol *via* pregnenolone and progesterone. Key steroidogenic enzymes involved include CYP11A1, 3β-HSD, CYP17A1, and 17β-HSD.

**Figure 3.**
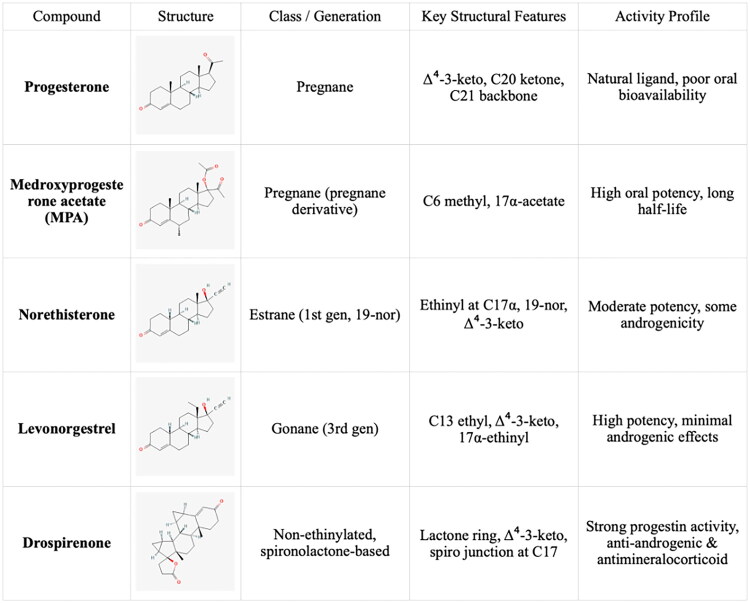
Comparative analysis of progestin classes and generations: Key structural features and activity profiles. The chemical structures for the described progestins are provided in the appropriate subsections.

Progestogens range from natural to synthetic progesterone molecules, presenting a nuanced classification system. The detailed classification is presented in Table 1. Notably, synthetic progestogens comprise a broad group known as progestins, characterized by their synthetic origin and varied pharmacological profiles [10,19]. Apart from this classification system, a traditional categorization divides progestins into old and new. These are divided into first, second, third and fourth generations. The first three generations are derivatives of testosterone (TE). They have an affinity for androgenic receptors (AR) and may cause undesirable androgenic effects. The fourth generation has activity similar to progesterone (P4) and therefore does not show androgenic effects like the first three generations. P4 is synthesized from cholesterol. Furthermore, P4 is a part of the TE biosynthesis pathway [21]. Figure 2 shows the conversion of cholesterol to P4 and TE. Table 2 contains full names and functions of the ensymes. Further classification based on structural properties, including mainly pregnanes, estranes, and gonanes, elucidates the intricate diversity within this pharmacotherapeutic domain [11]. A common feature of the progestogens’ chemical structure is the presence of a 3-keto group and a double bond between C4 and C5 in the A-ring, collectively referred to as the Δ4-3-keto group. If a given progestin lacks a Δ4-3-keto group, it is reached as a prodrug. It exclusively reaches its activity after oral administration, when it is transformed into the appropriate form. Therefore, depending on the position of the methyl group, progestins related to P4 are divided into pregnanes (methyl group at the C10 position) and norpregnanes (no methyl group at C10 or no C19). TE-related progestins can be divided based on the presence of an ethyl group at the C13 position. Based on the above, estranes (no ethyl group in C13) and gonanes (ethyl group in C13) are distinguished [21]. Figure 3 illustrates the chemical structure and structure-activity relationships (SARs) of progestins. Progestins share many common applications. However, despite their general classification, they exhibit varying levels of effectiveness in specific medical conditions [24].

**Table 1.** Classification of progestogens [21–23].

Progestogen		Agent	Generation
Progesterone		Natural progesterone	N/A
Retropogesterone		Dydrogesterone	N/A
Progesterone derivative		Medrogesterone	N/A
Pregnanes (17α-hydroxyprogesterone derivatives)		Medroxyprogesterone acetate	1
		Megestrol acetate	1
		Chlormadinone acetate	1
		Cyproterone acetate	1
Norpregnanes	17α-hydroxynorprogesterone derivatives	Gestonorone caproate	N/A
		Nomegestrol acetate	4
	19-norprogesterone derivatives	Demegestrone	N/A
		Promegestrone	N/A
		Nesterone	4
		Trimegestrone	4
Estranes (19-nortestosterone derivatives)		Norethisterone (Norethindrone)	1
		Norethisterone acetate	1
		Lynestrenol	N/A
		Ethinodiol acetate	N/A
		Norethinodrel	N/A
Gonanes (19-nortestosterone derivatives)		Norgestrel	2
		Levonorgestrel	2
		Desogestrel	3
		Etonogestrel	3
		Gestodene	3
		Norgestimate	3
		Dienogest	4
Spironolactone derivative		Drospirenone	4

Due to their chemical origin, progestogens are divided into progesterone, retroprogesterone, progesterone derivative, spironolactone derivative and several larger groups such as pregnanes, nonpregnanes, estranes and gonanes. Individual agents were assigned to corresponding groups. N/A: not applicable; 1: first generation; 2: second generation; 3: third generation; 4: fourth generation.

**Table 2.** Enzymes involved in the enzymatic pathway of steroidogenesis leading to testosterone synthesis.

Enzyme	Full Name	Function
CYP11A1	Cholesterol side-chain cleavage enzyme	Converts cholesterol to pregnenolone
3β-HSD	3β-Hydroxysteroid dehydrogenase	Converts pregnenolone to progesterone
CYP17A1	17α-hydroxylase / 17,20-lyase	Adds 17α-OH, then removes side chain
17β-HSD	17β-Hydroxysteroid dehydrogenase	Converts androstenedione to testosterone

The current study aims to undertake an exhaustive examination of existing literature pertaining to the utilization of progestogens in clinical practice. By synthesizing previous research findings and elucidating emerging perspectives, this review seeks to provide a comprehensive guide delineating the diverse applications and therapeutic considerations surrounding progestogens in contemporary medical practice.

## 2. Materials and methods

A literature review was conducted by PubMed, Google Scholar, Scopus and ClinicalTrials. The following search terms and their combinations were used when reviewing the aforementioned databases: (progestogen OR progestogens OR progestin OR progestins) AND (women OR woman OR men OR man OR adult). The analyzed publications about progestogens were dated from 2005 to 2024. The review process began in November 2023 and continued for 8 months until June 2024.

Initially, the list of titles and abstracts from the available papers was examined. Full-text publications about progestogens were used in the study. Books and documents, clinical trials, meta-analyses, randomized controlled trials and systematic reviews were included. Abstracts from conferences, reviews, case reports and articles not written in English were excluded. The chemical structures of the compounds we described come from PubChem.

## 3. Natural progestin - progesterone

Progesterone (P4) is an endogenous steroid hormone produced by the adrenal cortex, ovaries and testes. It is also produced by the ovarian corpus luteum during the first 10 weeks of pregnancy. After this time, it is secreted by the placenta. P4 has many functions in the human body, mainly in the reproductive system, maintains the endometrium during pregnancy by reducing uterine myometrial contractility and regulates uterine myometrial blood flow during ovulation [25–27].

P4 fulfils a role in maintaining pregnancy by regulating the immune response of the mother’s body, inhibiting inflammatory reactions, reducing uterine contraction and improving circulation in the uterine-placental area. Particularly in the early stages of pregnancy, progesterone has a key function in preparing the endometrium for embryo implantation and maintaining the gestational sac inside the uterus. It stimulates endometrial endothelial proliferation and increases the number of vessels, leading to increased blood flow [25,28].

Additionally, P4 has a crucial function in the menstrual cycle. During the luteal phase, it induces cell proliferation in the endometrium by leading to an enlargement in its thickness. This process causes the endometrium to become thicker, which in turn increases the surface area available for potential embryo implantation [25]. In addition to the above, P4 redounds to bone formation. It stimulates the growth of osteoblasts responsible for bone formation by the P4 receptor [25].

## 4. Progestins

### 4.1. Pregnanes

#### 4.1.1. Medroxyprogesterone acetate (MPA)

**Figure 4.**
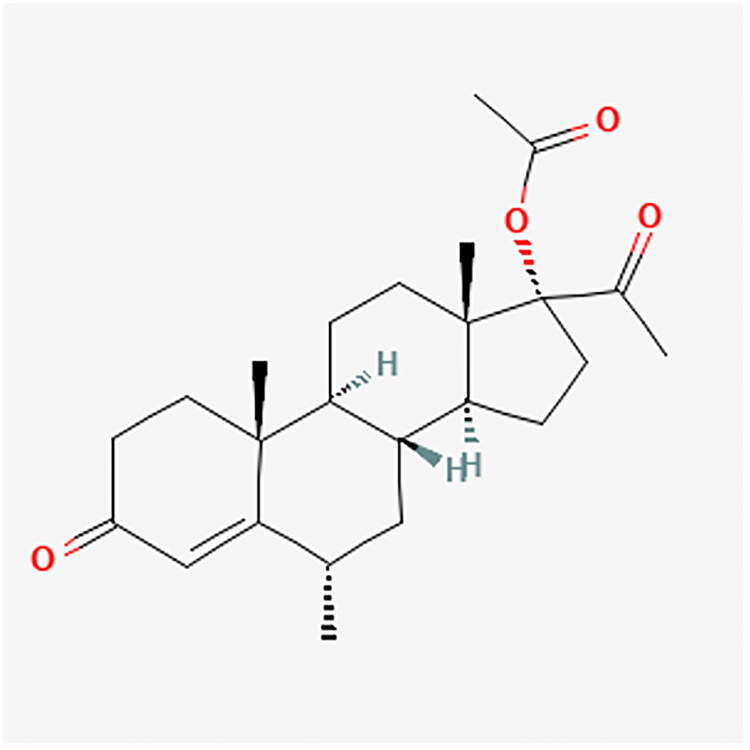
MPA chemical structure.

Treatment with MPA may be administered in several forms, including oral, intramuscular, and subcutaneous. In addition, MPA has a long-acting form termed depot medroxyprogesterone acetate (DMPA) [1]. Figure 4 shows the chemical structure of MPA. Due to its action, MPA is an oral agent used in ovarian stimulation therapy for *in vitro* fertilization (IVF) as a Progestin-Primed Ovarian Stimulation protocol (PPOS) [29]. Thereupon, it prevents moderate or severe ovarian hyperstimulation syndrome (OHSS) in women with regular ovulation as well as those with Polycystic ovary syndrome (PCOS), providing more embryos and a thicker endometrium [30–32]. Furthermore, it protects against premature LH surge during controlled ovarian hyperstimulation (COH) for IVF [33–35]. Studies have shown MPA may be used in women with endometriosis but with regular ovulation [29,36]. However, more oocytes are produced if MPA is combined with human menopausal gonadotropin (hMG). One study examined 450 patients with severe endometriosis undergoing IVF/Intracytoplasmic Sperm Injection (ICSI). Women were randomized to MPA + hMG, DYD + hMG, and P4 + hMG. Ovulation was induced using gonadotropin hormone-releasing agonist (GnRHa) and hCG. The number of oocytes collected in the MPA + hMG group was higher compared to DYD + hMG and P4 + hMG (9.3 vs. 8.0 vs. 7.6; *p* = 0.021). Moreover, pregnancy outcomes after frozen-thawed embryo transfer (FET) cycles were also compared in the individual MPA + hMG, DYD + hMG, and P4 + hMG groups due to clinical pregnancy and ongoing pregnancy (57.53 vs. 56.50 vs. 41.39). The results regarding live births were similar in the three groups [29].

MPA is an oral form of treatment for EH in women with IUD-related dysmenorrhea, anatomic factors complicating IUD placement or with a desire to have children [16,37]. However, there is a higher side effects risk including sedation, flatulence, nausea, headaches, mood swings, depression or irregular vaginal bleeding. Cyclic MPA therapy is frequently a cause of irregular bleeding compared to continuous treatment [37]. It is significant to take into consideration that the oral MPA does not provide contraception. Thereupon, oral MPA therapy has to be combined with an appropriate form of active contraception due to the risk of pregnancy [38].

DMPA is a long-acting form of MPA with intramuscular or subcutaneous administration. The advantages of DMPA as a contraception method compared to POP are a high rate of amenorrhea and long intervals between subsequent medication administrations [1,39]. The administration of DMPA is every 11–13 weeks [39]. Disadvantages include initially prolonged bleeding after the first dose, body mass increase and low bone density (BMD) [1]. To date, the DMPA subcutaneous implant has been shown as a proper male contraception in combination with TE [10]. Due to no effect on the milk composition, development of the infant and milk supply, DMPA is the hormonal contraceptive method in lactation. Nevertheless, no earlier than 6 weeks postpartum.

In addition, DMPA is a treatment in post-abortion women. Injection of DMPA should be offered on the same day as a medical abortion, especially in women experienced intimate partner violence. It does not affect the success rate of medical abortion with mifepristone and simultaneously gives positive effects of contraception starting from the first day after the procedure [40–42].

Contraindications of MPA application as oral and injection POC in menstrual suppression are BMD and risk factors of BMD, including intense physical exercise with weights, and a history of bone stress injury. Particular attention should be paid to patients with poor nutrition, middle age, chronic steroid users, and immobility. In addition, MPA is contraindicated in adolescents up to five years after menarche who have not yet reached peak bone mass [1]. According to one study, combining MPA with Conjugated Equine Estrogens (CEE) increases the risk of breast cancer. Therefore, the MPA and CEE combination is contraindicated in women aged 50–59 [43,44]. The above conclusions are confirmed by another study using MPA+CEE conducted on 16,608 postmenopausal women aged 50–79. The use of MPA+CEE was statistically associated (*p* < 0.001) with a higher incidence of breast cancer compared with the placebo group (annual incidence, 0.45% vs. 0.36%) [44].

#### 4.1.2. Megestrol acetate (MA)

**Figure 5.**
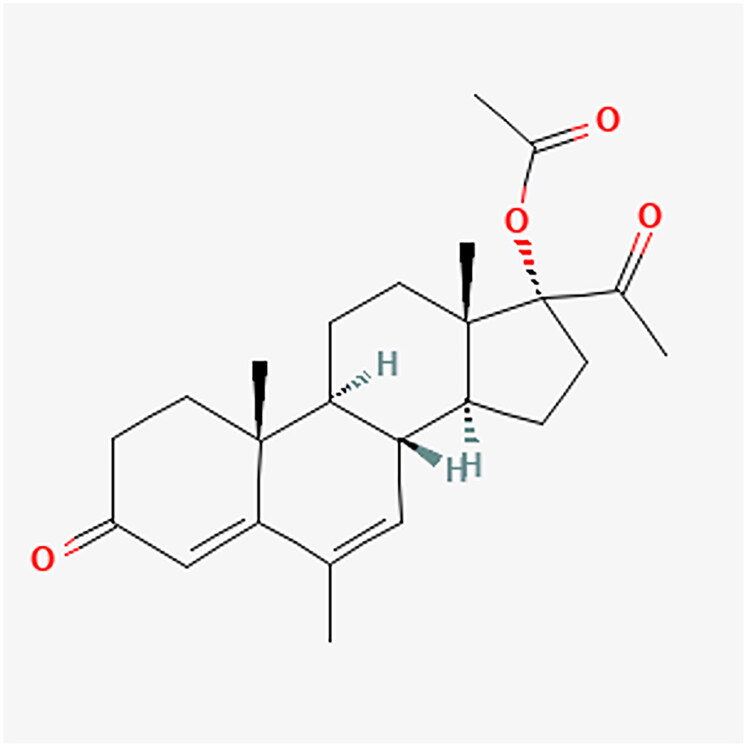
MA chemical structure.

By acting on various pathways in the organism, MA may be used in disparate conditions as a tablet or concentrated oral suspension form [4]. Figure 5 shows the chemical structure of MA. The study has shown that MA causes appetite stimulation in HIV/AIDS patients with massive weight loss as well as anorexia, cachexia syndrome and geriatric wasting syndrome [45]. The appetite stimulation occurs through the up-regulation of Neuropeptide Y levels and down-regulation of tumor necrosis factor α (TNF α), interleukin-1(IL-1) and interleukin-6 (IL-6). Therefore, there is fat tissue growth, a decrease in the degradation of muscle proteins, an increase in albumin levels, an increase in body weight and proper nutrition [4]. Anorexia-cachexia syndrome in cancer patients, as well as endometriosis, ovarian cancer and advanced prostate cancer, are off-label uses. Prostate cancer therapy is possible due to inhibiting 5-α-reductase and reducing dihydrotestosterone levels [46]. Moreover, low-dose MA reduces hot flashes in men who receive androgen suppression therapy for prostate cancer, as well as in menstrual women. Another use of MA is second or third-line palliative treatment in advanced endometrial and breast cancer by its suppression of LH and estrogen. In addition, it may be used as an alternative therapy in EH without atypia [4]. Equally, as mentioned above, MPA, oral MA is a fertility-sparing approach in patients with endometrioid EC [47].

Side effects of MA treatment include mainly weight gain and increased appetite. There is a risk of nausea, vomiting, rash, diarrhoea, vaginal bleeding, oedema, dyspnea, hypogonadism, hyperglycemia, fluid retention, loss of libido, hypertension, as well as thrombophlebitis and deep venous thrombosis as severe side effects [48]. Furthermore, MA, as a glucocorticoid agonist, may cause new-onset diabetes or worsen existing diabetes. Cushing-like symptoms and secondary adrenal insufficiency symptoms, including hypotension, fatigue and muscle weakness, are other severe glucocorticoid adverse effects [4,49].

Contraindications include a history or a high risk of thromboembolism and an active thromboembolic event. An absolute contraindication of MA therapy is pregnancy and breastfeeding. Breastfeeding postpartum women should not use COC-containing MA because of the risk of changes in breast milk amount and composition [4,50]. Moreover, in the group of patients with renal or hepatic impairment, there is a need for control during MA treatment [4].

#### 4.1.3. Cyproterone acetate (CPA)

**Figure 6.**
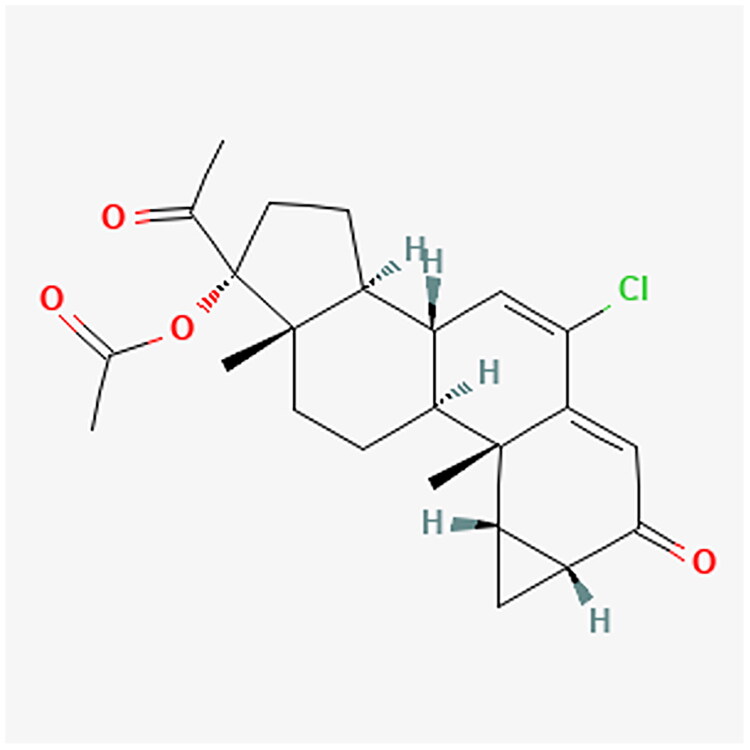
CPA chemical structure.

Due to its antiandrogenic effect, CPA is used in conditions with hyperandrogenism. It is available in oral and intramuscular form [51,52]. CPA competes with dihydrotestosterone for the AR. By binding to AR, it reduces androgen levels. Figure 6 shows the chemical structure of CPA. In addition, CPA inhibits the conversion of TE to dihydrotestosterone in the skin by lowering the activity of 5-α-reductase. Moreover, it reduces the secretion of gonadotropins and, consequently, the stimulation of the ovaries and testicles to androgen production. CPA is applied alone or in combination with estrogens [53,54]. It is a treatment in women with diagnosed PCOS to regulate menstrual cycles and reduce symptoms associated with hyperandrogenism [54]. According to studies, CPA in combination with estrogens is used for feminization in transgender women (TW) [53,55]. One study involved 64 people. They were divided into two groups based on the dose of CPA. The group with the lower dose of the drug included 32 TW treated with a dose of 10 mg/day and an additional 6 TW treated with a dose of 20 mg/day. The higher-dose group consisted of 25 TW treated with 50 mg/day and an additional 6 TW treated with 100 mg/day. Estradiol levels were adjusted to the CPA dose. Analysis showed no difference in TE suppression between the two groups. TE levels were suppressed or within the normal range for cisgender women (CW). At 6 months, 87% and 84.2% of patients had suppressed or normal levels for CT and at 12 months, 83.3% and 88.2% of patients in the low- and high-dose groups, respectively (*p* = 1 for both comparisons). Thus, at 12 months, TE levels were undetectable, within the normal range, or above the normal range for CW. However, it was below the lower limit of the normal range for cisgender men in 40%, 45.7%, and 5.7% of all patients. Side effects associated with CPA use include hyperprolactinemia, increased risk of meningioma, liver damage, and an increased risk of prostate cancer in men. A summary of the study is presented in Table 3 [53].

**Table 3.** A summary of the study with TW [53].

Group	Number of patients		Dose of CPA [mg/day]	Patients’ TE suppression [%]	
				At 6 months	At 12 months
Low-dose	38	32	10	87	83,3
		6	20		
High-dose	31	25	50	84,2	88,2
		6	100		

In addition to the above, CPA is the treatment of hypersexuality disorder. It performs by decreasing libido and reducing the incidence of sexual fantasies. It is a substance used in men with a paraphilic type of sexual disorder [56–58]. Further, CPA is a therapy for inoperable prostate cancer [52]. The contraceptive effect of CPA in men is presented in the subsequent paragraph.

### 4.2. Estranes: norethindrone/norethisterone (NET)

**Figure 7.**
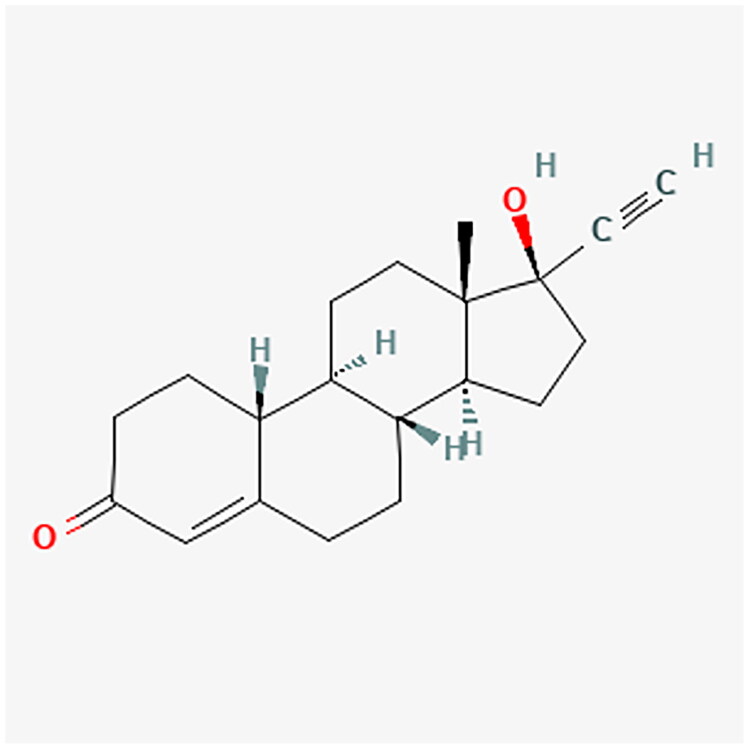
NET chemical structure.

**Figure 8.**
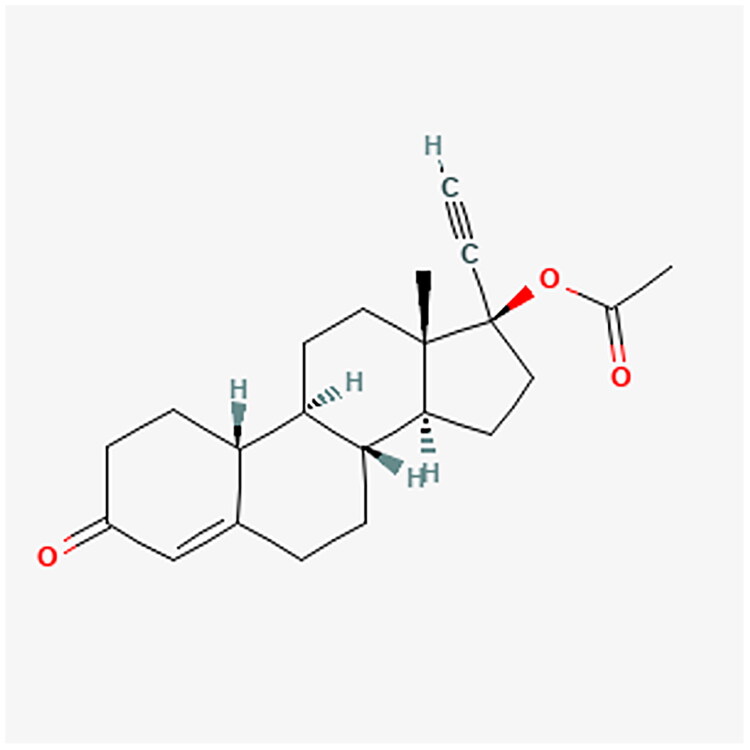
NETA chemical structure.

NET and norethindrone acetate (NETA) are widely used POPs, with their clinical applications varying based on dosage [1]. Figures 7 and 8 show the chemical structure of NET and NETA. These compounds are frequently employed in the management of endometriosis-related symptoms, including dysmenorrhea and abnormal uterine bleeding [59].

Relugolix, an oral GnRHa, is commercially formulated in combination with add-back therapy, consisting of estradiol and NETA. Its primary indication is the management of heavy menstrual bleeding associated with uterine fibroids, though its therapeutic scope is increasingly being extended to address endometriosis-related pain [60,61]. A pivotal result of the clinical trials from 2021 (LIBERTY 1 and LIBERTY 2) demonstrated the efficacy and safety of relugolix combination therapy in women with symptomatic uterine fibroids. The study reported significant reductions in menstrual blood loss and improvements in quality of life, with add-back therapy mitigating hypoestrogenic side effects [61]. In addition, Giudice LC et al. showed that this combination may be used to treat endometriosis-related pain. Improvement of dysmenorrhea and non-menstrual pelvic pain occurs after 4 weeks. The trial involved 638 women aged 18–50. They were randomized to a placebo group, a relugolix combination group (40 mg once a day, orally + estradiol 1 mg + NET 0.5 mg, orally) and a delayed relugolix combination group (40 mg in monotherapy, followed by 40 mg once a day, orally estradiol 1 mg + NET 0.5 mg, orally, each for 12 weeks). The dosing period was 24 weeks [60].NETA, when combined with estradiol, is also employed for the management of vasomotor symptoms in postmenopausal women, including hot flashes and sleep disturbances. A randomized, placebo-controlled trial showed that the combination of ultra-low-dose 17-β-estradiol (0.5 mg) and NETA (0.1 mg) significantly reduced the frequency and severity of hot flushes over a 3-week treatment period [62]. Additionally, it is considered safe for use in breastfeeding women, as it does not adversely affect milk production or the newborn. It may also help protect against BMD loss during lactation [3]. Furthermore, oral norethisterone has been studied for its efficacy in treating uterine arteriovenous malformations (AVMs), similar to MPA [63].

### 4.3. Gonanes

#### 4.3.1. Levonorgestrel (LNG)

**Figure 9.**
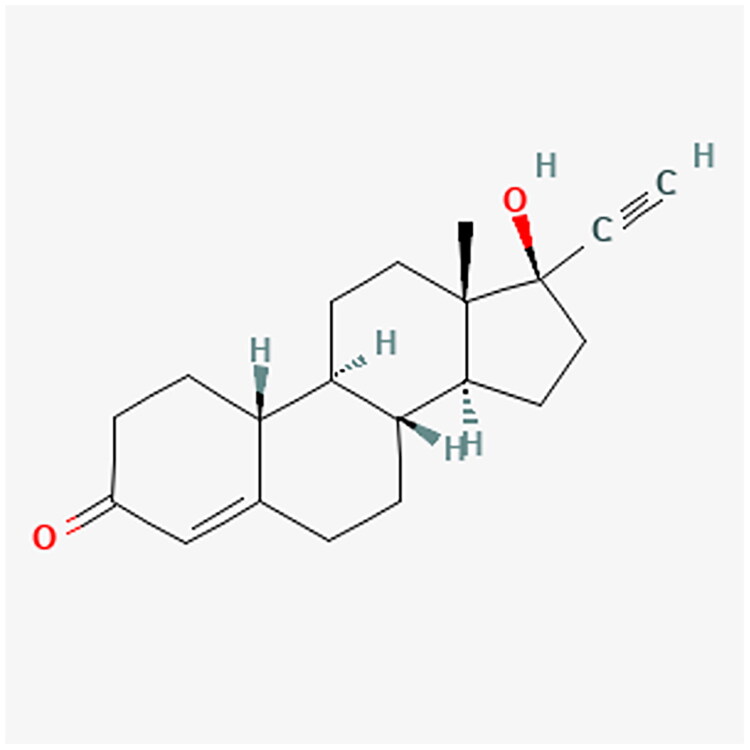
LNG chemical structure.

LNG, is a synthetic progestin and the active isomer of norgestrel racemide. Figure 9 shows the chemical structure of LNG. LNG is a commonly used effective Long-Acting Reversible Contraceptive (LARC) in the form of subcutaneous implants and the LNG-IUD [1,3]. In the realm of exchange contraception, LNG stood as a pioneering treatment. The mechanism of LNG action includes inhibition of the LH and FSH secretion. It leads to inhibition of ovulation, reduced transport of oocytes through the fallopian tube, and impeded embryo implantation through the maladaptation of the endometrium. The main disadvantage of this method of contraception is occasional breakthrough bleeding, which may occur in the first 3–6 months of use [1]. There may also be potential side effects such as mood changes, headaches, skin reactions (including acne), dizziness, an increase in weight, breast discharge and transient ovarian cysts [40]. There are contraindications to using LNG, such as irregular menstrual bleeding, active liver disease, pregnancy, breast cancer, cancer of the genital tract and cardiovascular disease. Studies reported that in users of the 2-rod system, those with a bodyweight exceeding 70 kg exhibit LNG concentrations around 45% lower than those weighing under 50 kg, with a decrease in mean serum levels correlating to increasing body weight by approximately 0.0033 μg/L/kg [64,65]. Another fact is that the efficacy of LNG is inversely proportional to a patient’s BMI, where the higher the BMI, the lower the efficacy [66]. Compared to the use of oral contraceptives, the rate of ectopic pregnancies is lower, at about 1.47 per 1000 users of LNG [67]. If a woman waits to remove the implant after 5 years, there is still 69% of the substance in the capsules that provides some contraception. Thus, the implant can remain inside the uterus for a long time. However, after implant removal, fertility usually returns within 3 months in 50% of people, and within 1 year in 80% [40]. One of the advantages of LNG-IUD is that it can be used in young patients, making it an attractive option for adolescents [1].

One of the studies suggests that the use of oral contraceptives containing ethinylestradiol along with LNG may affect breast milk composition, manifesting as reduced milk volume and a decrease in protein, lipids, calcium and phosphorus [68]. Another study has reported that newborns of breastfeeding mothers who received LNG may present decreased thyroid hormone values. However, no other negative effects affecting the baby’s health have been observed [69]. Studies indicate that this form of contraceptive treatment has the potential to protect the mother’s bones during breastfeeding, or at a minimum, not to worsen the problem of BMD [70–72]. Even when it is taken after intercourse (a postcoital method), LNG does not negatively affect breastfeeding or the baby’s health. Being a synthetic progestogen and the active isomer of norgestrel, LNG emerges as a secure and efficient contraceptive choice for breastfeeding mothers [3,67,73].

Regarding women within 48 h to less than 4 weeks after childbirth, the use of a uterine insert is usually not recommended [74]. Some other guidelines allow delayed insertion of IUDs after childbirth if immediate insertion is not possible [75]. The use of an LNG-IUD is not recommended if there are anatomical irregularities in the uterus, such as a septate or bicornuate uterus, ongoing genitourinary infections, or if the individual has current breast, endometrial, or cervical cancer [76,77].

Increasingly, the LNG-IUD is being used as first-line therapy in EH. The IUD emits LNG throughout 4 to 5 years, proven to be an effective therapeutic agent. Research reports that as many as 90% of patients who present with benign EH achieve a return to a healthy endometrium [78,79]. As well, complete regression of lesions is observed in 75% of patients with atypical or early EC [80]. However, treatment of EC involves a comprehensive approach [37].

#### 4.3.2. Norgestrel (NG)

**Figure 10.**
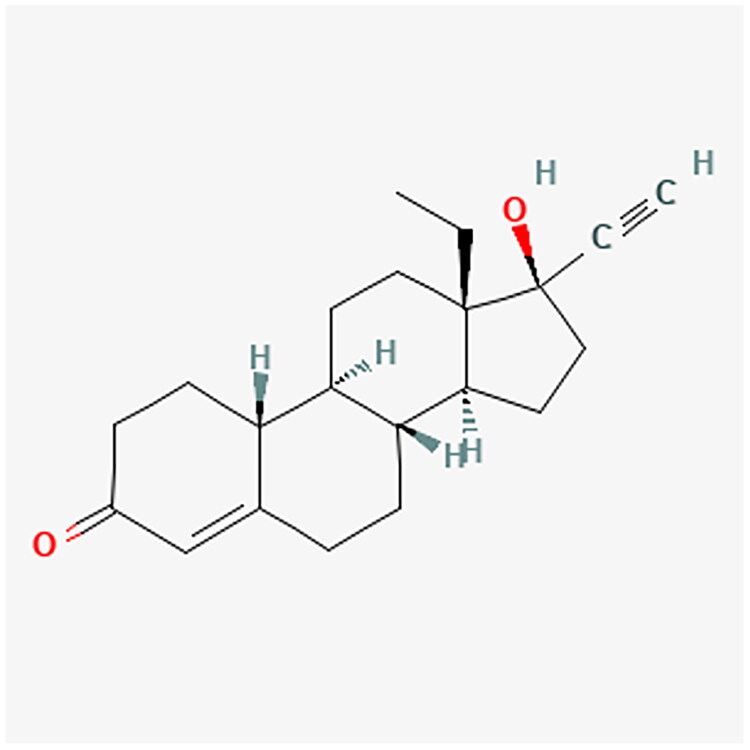
NG chemical structure.

NG is a POP that has been used as a contraceptive method. The mechanism of action of NG involves binding to specific carrier proteins in plasma, similar to the sex hormone-binding globulin (SHBG). It primarily works by inhibiting ovulation and has been used in various contraceptive methods, including oral pills and subdermal implants [81]. Figure 10 shows the chemical structure of NG.

Breast cancer is a contraindication to all hormonal therapies, including NG. Individuals with current breast cancer or a history of breast cancer in remission for less than five years should avoid using NG. Additionally, those with cirrhosis, benign liver tumors, or ischemic heart disease are advised against using NG due to potential risks [82,83].

D-NG’s affinity for plasma carrier proteins, such as TE-binding globulin, contributes to its androgenic effects, potentially leading to increased acne development in women. The fluctuation in plasma levels after daily oral administration may exacerbate these effects by releasing peaks of biologically active D-NG, displacing TE. Common side effects of NG also include menstrual bleeding irregularities, headache, gastrointestinal disturbances, mood changes, and dysmenorrhea [81].

For both breastfeeding and non-breastfeeding women, NG proves highly effective for contraception. In non-breastfeeding women, NG monotherapy has an overall Pearl index of 2.2 [83–85]. In breastfeeding women, individual studies report low pregnancy rates, with the largest study showing a pregnancy rate of 1.2 per 100 women with perfect use [84,86–90]. Upon conclusion, a total of 2,202 women had employed NG 75 µg/day for an average duration of 13 months [84]. Incorrect or inconsistent use of the contraceptive pill accounts for more than half of pregnancies in breastfeeding women [90].

Beyond its contraceptive applications, NG has been detected in environmental matrices, indicating its presence outside medical use [91]. Additionally, it has been researched in the mouse retina as a neuroprotective agent for the treatment of retinitis pigmentosa, a retinal disease, showing promise in preserving vision [92].

#### 4.3.3. Desogestrel (DSG): etonogestrel (ENG)

**Figure 11.**
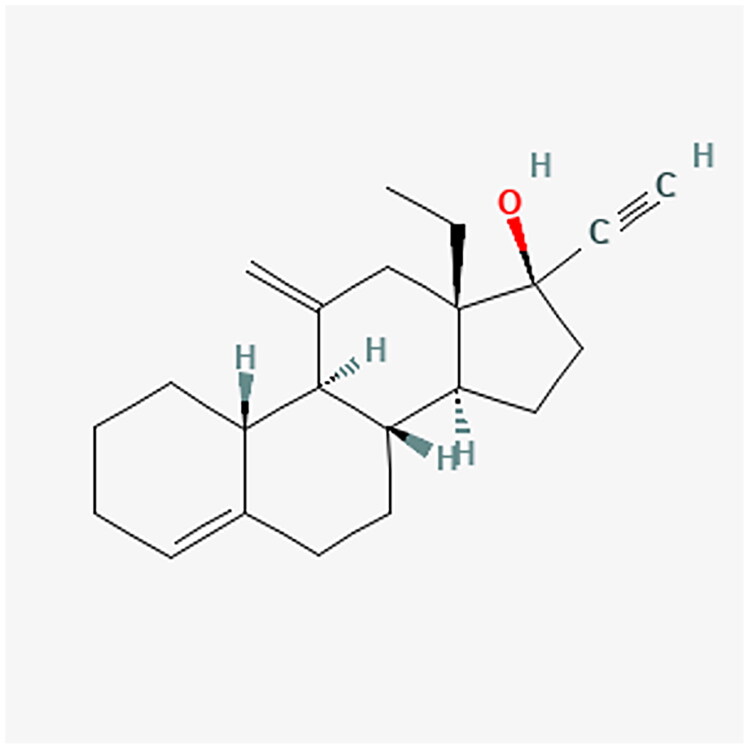
DSG chemical structure.

**Figure 12.**
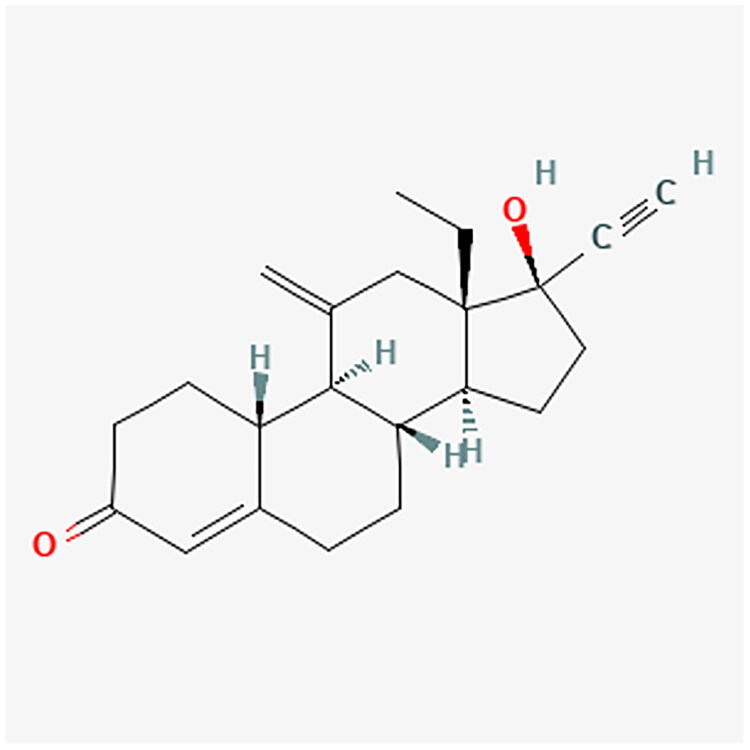
ENG chemical structure.

ENG is the main metabolite of DSG and has higher progestogen activity as well as lower affinity for androgen receptors than other progestogens. While DSG is commonly used in oral contraceptives, its metabolite, ENG, is the focus here due to its role in contraceptive implants and intravaginal rings. Figures 11 and 12 show the chemical structure of DSG and ENG. Its main mechanism of action in contraception is to inhibit ovulation by preventing the release of LH from the pituitary gland. Furthermore, ENG thickens cervical mucus, making it difficult for sperm to enter the uterus, and modifies the uterine lining, preventing the implantation of a fertilized ovum [93–95].

As a subcutaneous contraceptive implant, ENG demonstrates remarkable contraceptive efficacy, achieving rates of up to 100% during therapy [96–98]. The implantation of ENG is mainly recommended for women of reproductive age, from 15 to 45, who are not pregnant. Two types of contraceptive implants containing ENG are available: a first-generation implant (non-radioactive and non-trackable) and a second-generation implant (radiopaque due to the inclusion of barium sulfate, allowing it to be visualized on X-rays). Both contain 68 mg of ENG and are effective for up to 3 years. The second-generation implant is described as non-teratogenic, posing no risk to the fetus, though such data are not explicitly reported for the first-generation implant. Both implants can cause different side effects and complications specific to ENG [40,93,99].

Moreover, ENG in combination with ethinyl estradiol (EE) is used in the intravaginal contraceptive ring method. The ring is placed in the vagina and releases both components regularly over a specified period, usually through the menstrual cycle. It is also an effective method of contraception that eliminates the need to take birth control pills daily [100]. Studies reported that the ENG/EE ring with the Pearl Index was from 0.65 to 1.18 [101,102]. The ENG/EE ring is inserted into the vagina and stays in place for 3 weeks continuously. After removal for 1 week to allow for withdrawal bleeding, a new ring is inserted [100].

Contraindications for ENG include a history of liver disease and breast cancer. It is not recommended for use in pregnant women. Compared with CHC, which are contraindicated in women with a history of deep vein thrombosis (DVT) because of their association with an increased risk of thromboembolic events, progestin-only methods, such as ENG implants, do not present an increased risk and are generally considered safe for use in this population. It does not affect the composition of milk, and the time of lactogenesis did not differ significantly between the studied groups [103–107]. Breastfeeding women should not use the contraceptive ring for the first three weeks after delivery due to an increased risk of deep vein thrombosis. In addition, COC should be avoided for the first 4 weeks after delivery and 6 weeks in women with risk factors for venous thrombosis [94].

Side effects associated with the ENG implant may include menstrual irregularity and intermittent bleeding between periods, known as breakthrough bleeding [1]. In a distinct examination, results showed that with every 100 pg/mL surge in serum ENG concentration, individuals utilizing the implant demonstrated a 1.6-fold increased probability of encountering abnormal bleeding and were 2.3 times more likely to receive a prescription for managing persistent bleeding [108]. The bleeding associated with the implant is unpredictable, leading up to 11% of users to discontinue it within the first year due to irregular bleeding [109]. There are also single cases of galactorrhea after implantation, although this usually resolves once the implant is removed. Women using ENG may experience slight weight gain, as well as unpredictable mood changes. In rarer cases, changes such as weight gain in male infants, or the incidence of respiratory ailments and skin problems in children of mothers using this type of contraception, may occur while nursing a child. Nevertheless, most of these side effects are statistically insignificant, so ENG appears to be relatively safe for breastfed infants. The research shows no significant effect on quantity or milk composition [94]. However, there are some reports of delayed secretory activation in women using this type of contraception [110].

Renowned primarily for its role in contraceptive implants, ENG offers a diverse array of non-contraceptive applications as well, positioning it as an exceptionally appealing therapeutic option. One of its main advantages is that it can be used in women with diabetes and hypertension. In addition, it is a safe therapeutic option for patients with cutaneous lupus erythematosus, Hashimoto’s encephalopathy and patients with a history of parenchymal organ transplantation. For gynecological diseases such as endometriosis, ENG is also showing promising results [40,59]. Research reports its effectiveness in reducing pain associated with endometriosis, particularly for symptoms associated with rectovaginal endometriosis and symptoms like pelvic pain, deep dyspareunia, dysmenorrhea and dyschezia [111–114]. In addition, a reduction in discomfort associated with endometrial cysts and regression of endometrial intraepithelial neoplasia were observed. ENG implants provide pain relief and also reduce the intensity of menstruation in patients with endometriosis, further improving the comfort of these women’s lives [40,59].

#### 4.3.4. Norgestimate (NGM)

**Figure 13.**
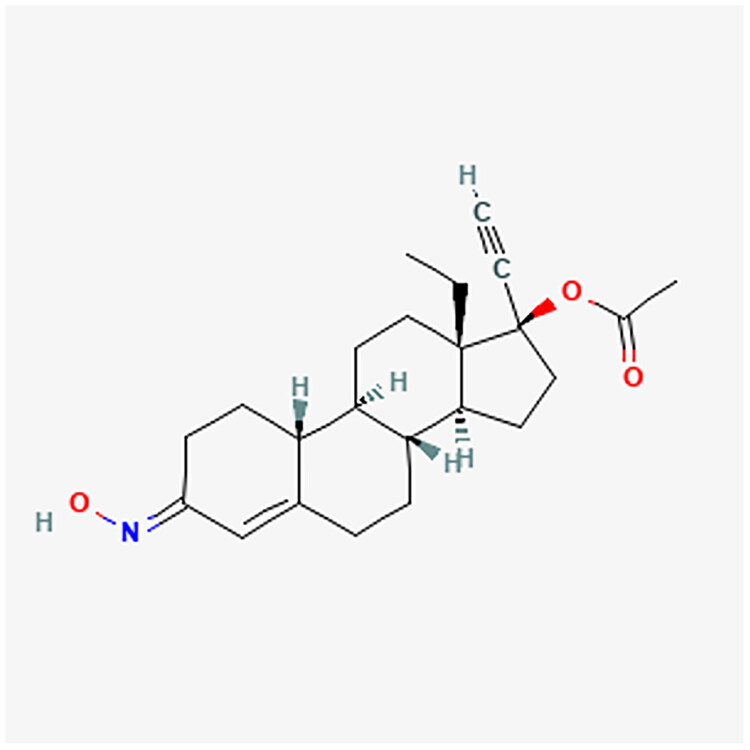
NGM chemical structure.

NGM is a progestin used in combination with EE as an oral contraceptive and has an excellent low Pearl Index of 0.25 [115]. It exhibits pharmacologic activity characteristic of a progestin, such as stimulating the endometrium and inhibiting ovulation by abolishing the preovulatory LH peak. It also alters cervical mucus to prevent sperm penetration and renders the endometrium incompatible with implantation [115–117]. It provides reliable cycle control with a low incidence of breakthrough bleeding and spotting [118]. NGM is a progestin that is rapidly absorbed and metabolized when taken orally. Unchanged NGM is not found in urine, indicating its complete metabolism [119]. Figure 13 shows the chemical structure of NGM.

NGM has minimal androgenic activity due to its low affinity for AR. In combination with EE, it also has favorable effects on lipid metabolism, including an increase in HDL cholesterol and a reduction in the LDL/HDL ratio, with minimal effects on carbohydrate metabolism. NGM/EE significantly elevates SHBG levels, further indicating low androgenicity, and shows no adverse effects on prolactin or coagulation. Some studies report improved acne and minimal weight gain. While its metabolic profile suggests reduced cardiovascular risk, long-term epidemiological data are needed to confirm these benefits, particularly in high-risk populations [115,120].

Some reported side effects associated with NGM include headache, nausea, mastodynia, and dysmenorrhea [7]. The study reported that the prevalence of acne in NGM pill users is 2% [121]. In a single study, there was a non-significant rise in CRP levels from 4.9 mg/l to 5.6 mg/l (*p* > 0.05) following the use of a combination including EE and NGM [122]. However, the incidence of these side effects is low, and they are not a common reason for discontinuation of the medication [123].

#### 4.3.5. Dienogest (DNG)

**Figure 14.**
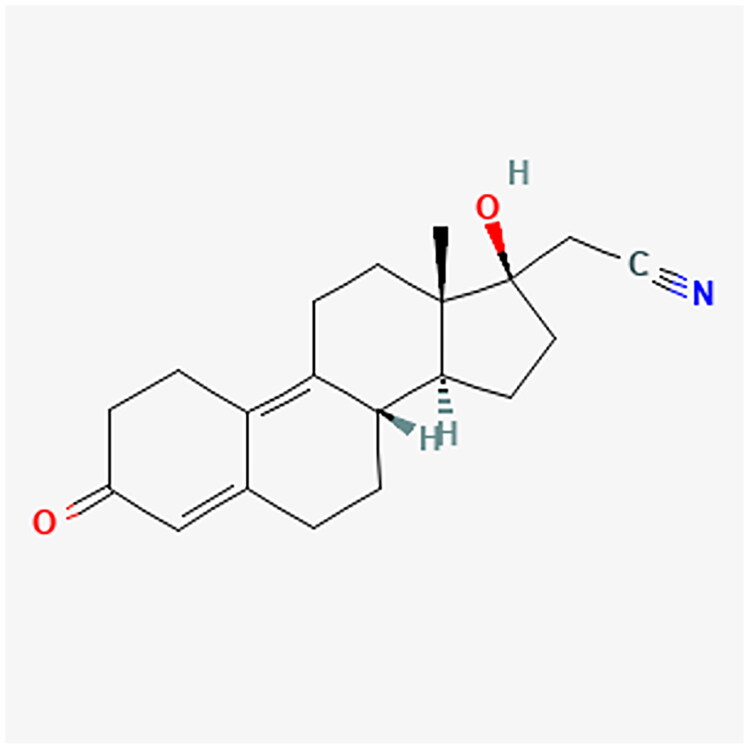
DNG chemical structure.

DNG is a fourth-generation selective progestin with concentrated oral activity. It is characterized by P4 action. Furthermore, it has minimal androgenic and estrogenic impacts. Due to its anti-angiogenic and anti-inflammatory properties, it reduces the activity of endometrial lesions through the local effect of P4 domination over estrogens [124,125]. Figure 14 shows the chemical structure of DNG. DNG generates minor side effects related to the hypoandrogenic impact. Compared with GnRHa, it causes spotting and weight gain but decreases hot flashes and the incidence of vaginal dryness. Hence, it can be used to treat pelvic pain caused by endometriosis. Pain conditions resolve within 6 months from the beginning of the treatment [124–127]. Moreover, one study showed that DNG may be a substitute for GnRHa in suppressing endometriosis before ovarian stimulation in IVF [128]. Correspondingly, it delays the recurrence and relieves the symptoms of endometrial cysts after fertility protection surgery [129]. Compared to GnRHa, by using DNG, women’s periods appear considerably faster. Thus, it allows them to become pregnant in a shorter time after the end of treatment [130]. The use of DNG as a COC with estradiol valerate (E2V) is known as a therapy against heavy menstrual bleeding and, consequently, as improving the patient’s quality of life and haemoglobin level [5,12]. Additionally, the effectiveness of DNG in dysmenorrhea treatment has been noticed, both when used alone and in the form of Low-Dose Estrogen-Progestins (LEP) [131,132].

### 4.4. Dydrogesterone (DYD)

**Figure 15.**
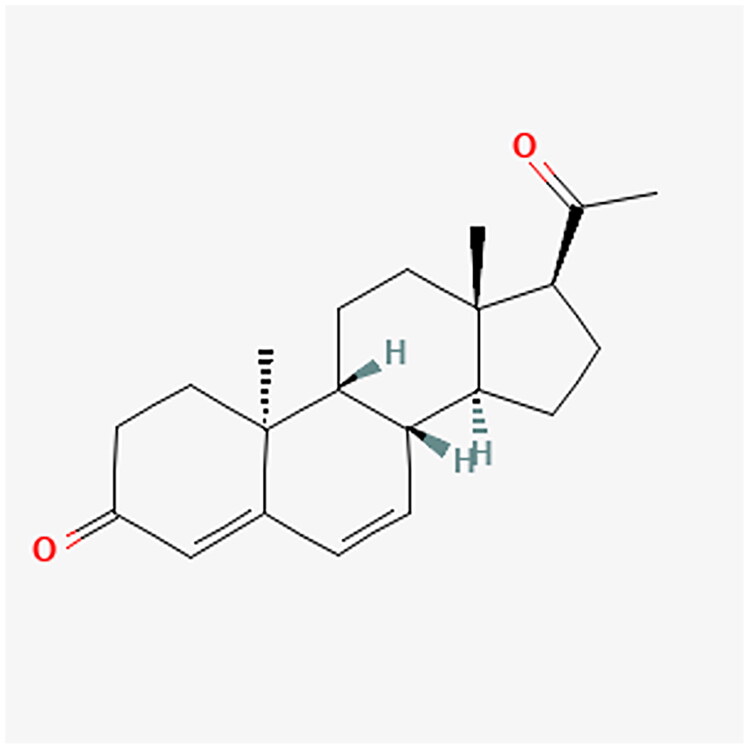
DYD chemical structure.

DYD is an oral reversal of P4. It is characterized by high selectivity towards P4 receptors and better bioavailability than micronised vaginal progesterone (MVP). Figure 15 shows the chemical structure of DYD. It is more effective at lower doses. DYD does not cause hormonal changes in patients or fetuses. DYD is used to treat various conditions associated with P4 deficiency, such as endometriosis, menstrual disorders and miscarriages [133–135].

DYD may relieve symptoms in endometriosis by inhibiting angiogenesis and proliferation of ectopic endometrium by decreasing the level of vascular endothelial growth factor (VEGF), CA125, E2, IL-6 and TNF-α [136,137]. Furthermore, DYD supports the luteal phase in FET, which improves IVF results. Additionally, the effectiveness of DYD in miscarriages treatment has been noticed. The study showed that the duration of recurrent contraction in participants who had recurrent preterm labor was significantly longer in the DYD group. Despite this, gestational age at delivery, pregnancy outcomes, and neonatal outcomes did not differ between the two groups [138–149].

### 4.5. Drospirenone (DRSP)

**Figure 16.**
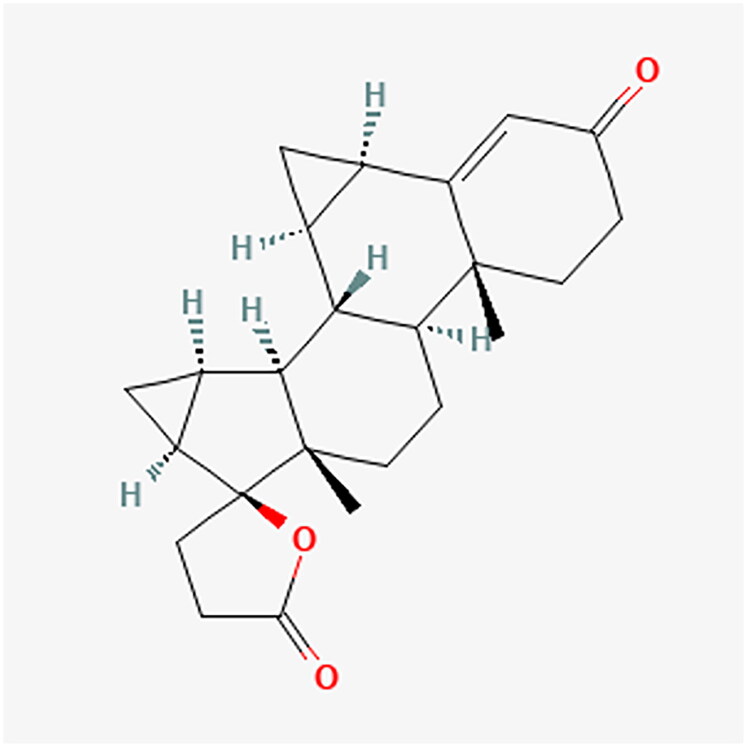
DRSP chemical structure.

As an analogue of spironolactone, DRSP is applied in oral form. In human metabolism, DRSP has antiandrogenic and antimineralocorticoid effects. Figure 16 shows the chemical structure of DRSP. Moreover, no impact on the breastfed infant or milk supply occurs [3]. In connection with the mentioned, DSP as a POP is preferred in breastfeeding women over COC with EE in 4 weeks postpartum. It is applied in patients with estrogen use contraindications for menstrual suppression. There are disadvantages to using POP, such as a low rate of amenorrhea and irregular breakthrough bleeding [1,3].

## 5. Anti-inflammatory and anti-angiogenic effect of P4

**Figure 17.**
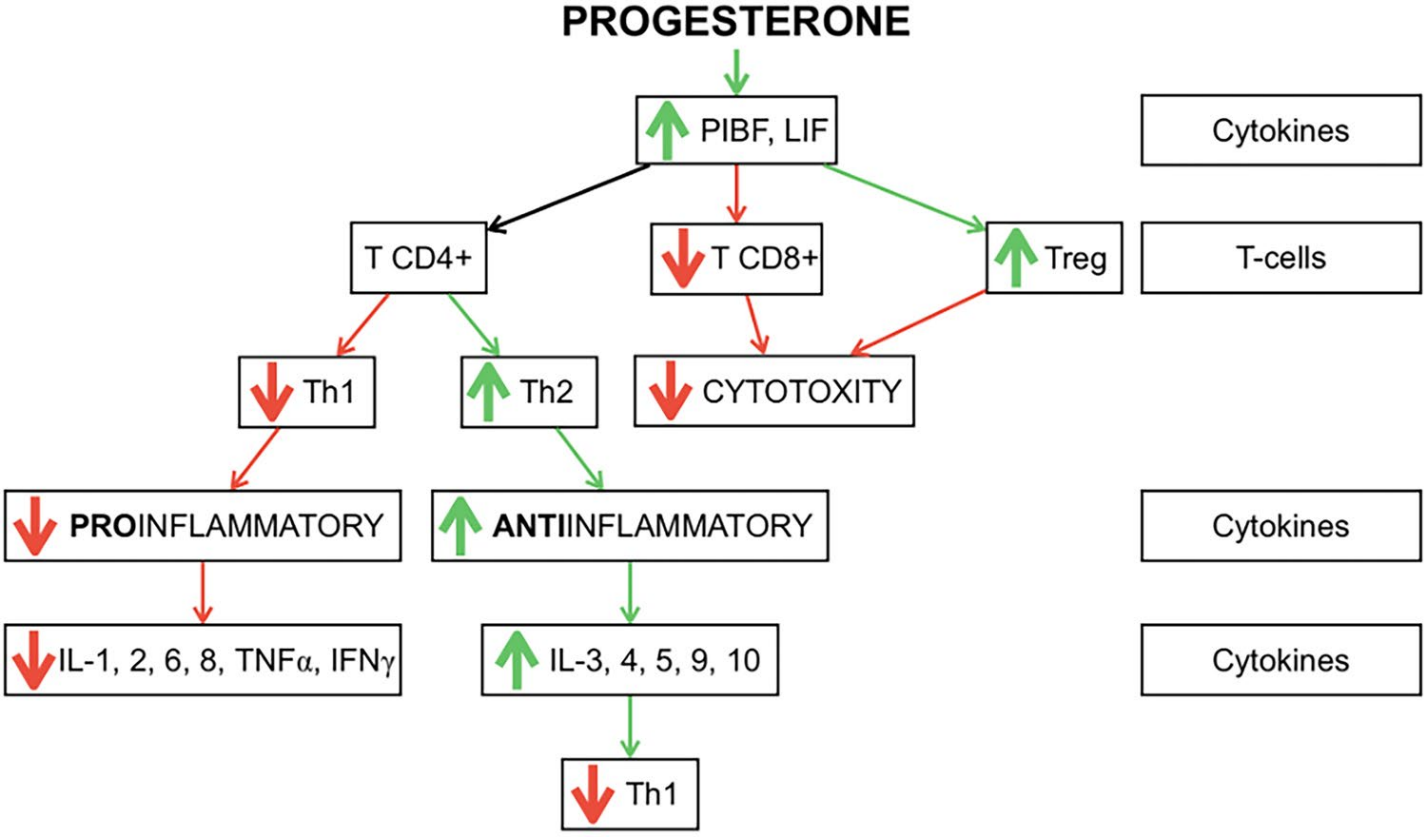
Mechanisms of P4 anti-inflammatory action [17,152–155]. green arrow - stimulation; red arrow - inhibition.

P4 has anti-inflammatory and anti-angiogenic effects [59,150,151]. The anti-inflammatory effect of P4 is based on its influence on cellular and humoral immunity. P4 regulation is associated with mediators such as P4-induced blocking factor (PIBF) and leukaemia inhibitory factor (LIF) [152]. PIBF is a P4-regulated gene and can be secreted by immune cells and host cells possessing the P4 receptor [153]. The increased level of this factor leads to an increase in the synthesis of cytokines responsible for immunotolerance, as well as to the introduction of cytotoxic lymphocytes themselves into a state of immunotolerance. Interestingly, CD4+ and CD8+ T cells show a dose-dependent effect of P4 [152]. LIF belongs to the IL-6 family of cytokines [154]. P4 increases LIF levels by increasing IL-4. LIF has anti-inflammatory and analgesic effects by reducing the expression of IL-1β and nerve growth factor (NGF) [151]. Both PIBF and LIF directly influence the differentiation of T lymphocytes. Figure 17 shows the mechanisms of immunomodulatory action of PIBF and LIF dependent on P4. The above-mentioned cytokines have an inhibitory effect on Th1 and reduce the production of pro-inflammatory cytokines. Moreover, they increase the differentiation of regulatory T lymphocytes (Tregs), which further enhances immune tolerance [155]. In addition to their antagonistic effect on Th1, they activate Th2 and thus increase the production of appropriate anti-inflammatory cytokines. Furthermore, they reduce CD8+ T cytotoxicity [17].

## 6. Progestins in combination with other substances

Considering the impact on tissues, progestins can be used in combination with estrogens as a COC in women. The most often applied estrogens are EE, esterol, estradiol, and mestranol. Nonetheless, there are combinations with E2V [1,11, 12,50,156]. Table 4 characterizes the possibility of using particular groups of progestins in the form of POC and COC.

**Table 4.** Possible combinations of progestins application as POC or COC in women [1–83].

Type	Progestins	POC	COC
Pregnanes	MPA	+	N/A
	DMPA	+	N/A
	MA	N/A	N/A
	CPA	N/A	+
Estranes	NET	+	+
Gonanes	LNG	+	+
	DSG: ENG	+	+
	NG	+	+
	DNG	+	+
	NGM	N/A	+
DYD		+	+
DRSP		+	+

MPA: Medroxyprogesterone acetate; DMPA: depot Medroxyprogesterone acetate; MA: megestrol acetate; CPA: cyproterone acetate; NET: Norethindrone; LNG: levonorgestrel; DSG: desogestrel; ENG: etonogestrel; NG: norgestrel; DNG: dienogest; NGM: norgestimate; DYD: dydrogesterone; DRSP: drospirenone; N/A: not applicable.

In addition to the case of using progestins with estrogen in COC, there is the possibility of combining them in MHT [12]. One of the main functions of progestins in MHT is to protect the endometrium from the proliferative effects of estrogens in women with an intact uterus, thereby reducing the risk of EC. In one study, women received daily CEE with MPA or placebo for an average of 5.6 years. After 13 years of observation, there were fewer cases of EC in the CEE+MPA therapy group compared with the placebo group (66 vs. 95 patients, annual incidence, 0.06% vs. 0.10%; hazard ratio [HR] = 0.65, 95% confidence interval [CI] = 0.48 to 0.89, *p* = .007) [157]. To date, studies have shown that the combined use of these substances in postmenopausal women improves the quality of sleep [13,158–162]. In one of the above-mentioned studies on sleep quality, 3,123 women aged 77–99 were included. In this group, patients were current MHT users, 1289 had used MHT in the past, and 1410 had never used MHT. Women currently using MHT were less likely to wake up during sleep compared to women who had never used this therapy. Current users had fewer long-wake episodes ≥ 5 min (6.5 vs. 7.1, *p* = 0.004) as well as less wakefulness after sleep onset (WASO) time (76 vs. 82 min, *p* = 0.03) than never users. Moreover, it was shown that past MHT users had longer total sleep time compared to women who had never used MHT (413 vs. 403 min, *p* = 0.002). Additionally, never users had significantly greater odds of ≥8 long-wake episodes (*p* = 0.002) and awakening after sleep onset ≥90 min (*p* = 0.03) than current MHT users. These patients also had higher odds of low sleep efficiency <70% and napping ≥2 h [158]. Additionally, COC MHT has a positive effect on hot flashes. One study describes the effect of oral combination therapy with ultra-low-dose 17β-E (0.5 mg) and DYD (2.5 mg) on the number of daily hot flashes and the number of moderate to severe hot flashes The study included non-hysterectomized postmenopausal women between the ages of 45 and 65 who had the last menstrual bleeding occurred at least 12 months before screening. All qualified participants experienced at least 50 hot flashes in total on seven consecutive days during the two-week screening period. The reduction in the number of daily hot flashes was more pronounced in the group receiving E 0.5 mg/D 2.5 mg than in the placebo group (mean difference was −1.5; 95% confidence interval: −2.1 to −1.0; *p* < 0.001). Patients on therapy also reported improvements in health-related quality of life, including relief of psychological symptoms and reduced vaginal dryness, as well as a high rate of amenorrhea [163].

The use of DNG as a COC combined with E2V is known as a therapy against heavy menstrual bleeding and, consequently, as improving the quality of life and hemoglobin level in patients [5,12]. However, regarding the use of DNG in breastfeeding women in the first 4 weeks postpartum, POC is a more suitable choice than COC [164]. There are also known different ways of using progestins as a male contraception, which will be discussed in the following part of the paper.

## 7. Progestins in oncology

According to the studies, the treatment of P4-sensitive cancers such as endometrial, renal and breast cancer involves the use of progestins [11,17–19, 165–167]. They may be an effective therapeutic option for patients with advanced, recurrent endometrial cancer (with low toxicity and positive P4 receptor), as well as in the treatment of deep endometriosis [168–175]. Reports also suggest that some progestins may be used in the treatment of hepatocellular carcinoma or liver metastases [129,176]. In addition, progestogens such as DMPA or NET can be used to treat uterine AVMs, according to the study. In the mentioned treatment, 6 classes of agents were applied: progestogens (57 patients), GnRH-a (28 patients), methotrexate (11 patients), COC (7 patients), uterotonic drugs (6 patients) and danazol (3 patients). Success rates were compared and showed high efficacy of progestogens (OR, 82.5%; 99% CI, 70.1%–90.4%; *p* < .001) as well as GnRH-a (OR, 89.3%; 99% CI, 71.4%–96.5%; *p* <.001) or methotrexate (OR, 90.0%; 99% CI, 55.8%–98.8%; *p* = .028). Moreover, it was reported that the complication rate associated with progestogen therapy was 10% compared to GnRH-a therapy (10.7%) and methotrexate therapy (18.2%) [63].

Another example of the use of progestins in oncology is MPA therapy, which is a fertility-sparing treatment for women with stage IA (FIGO) endometrioid EC. The study analyzed a group of 84 patients between the ages of 13 and 85 who were diagnosed with stage IA G2 EC and qualified for fertility-preserving treatment. The therapy included oral MPA, LNG-IUD and MA. Among the participants, 16 received a combination of MPA and LNG-IUD, 29 were treated with LNG-IUD alone, 14 received MA, and 16 received a combination of MA and MPA. Analysis of the results focused on oncological and reproductive aspects. 22 women became pregnant after the end of hormone therapy, with the majority conceiving naturally. In five cases, assisted reproductive technology (ART) was required. These results indicate the possibility of successful fertility-sparing treatment in women with stage IA G2 EC [47]. To date, guidelines for infertility therapy have only addressed EC in stage IA G1 [15]. However, there is an opportunity to introduce new strategies for identifying patients with potential for fertility preservation. The new molecular classification announced by the European Society of Gynecological Oncology (ESGO), the European Society of Radiotherapy and Oncology (ESTRO), and the European Society of Pathology (ESP) consensus in 2021 provides a basis for improving the treatment of patients with endometrial cancer, including fertility preservation [177].

In addition, progestins are oncologically used as LNG-IUDs in the treatment of complex atypical hyperplasia (CAH) and early low-grade EC. The study involved 46 patients treated with LNG-IUD. 15 patients were diagnosed with CAH, 9 had G1 EH, and 8 had G2 EH. The overall response rate after six months was 75% (95% CI 57–89), with an 80% response rate among patients with CAH, a 67% response rate among those with G1 cancer, and a 75% response rate among those with G2 cancer. The results showed that a larger uterus was associated with lower treatment efficacy (*p* = 0.04). The results suggest that LNG-IUD may be an effective conservative treatment option for patients with CAH or early low-grade EH [160].

Another study compared reports of progestational therapy as a preventive method for endometrial intraepithelial neoplasia. The reports included LNG-IUD therapy or oral progestins. Interestingly, there is a possibility of using the above methods as an alternative to surgical treatment. The study included 824 premenopausal patients. Each woman was diagnosed with endometrial intraepithelial neoplasia. The patients were divided into two groups. The first group consisted of 459 patients who received oral progestin as treatment. The second group consisted of 365 patients who used LNG-IUD. The response to treatment was assessed after 12 months. The response ratio to oral progestin at 12 months was 0.82 (95% CI = 0.69 to 0.91), and LNG-IUD was 0.95 (95% CI = 0.81 to 0.99). For both oral progestin (I2 = 58%, *p* < 0.01) and LNG-IUD (I2 = 79%, *p* < 0.01), there was significant between-study heterogeneity [159]. Table 5 shows summary of above clinical studies.

**Table 5.** Summary of selected clinical studies evaluating the use of various progestins.

Substance	Number of patients	Tumor type	Study outcomes
DMPA, NET	57	AVM	High efficacy: 82.5% (*p* < .001); low complication rate (10%)
MPA, LNG-IUD, MA	84	Stage IA G2 EC	22 pregnancies post-treatment (mostly natural); promising fertility-sparing therapy
LNG-IUD	46	CAH, G1 & G2 EC	Overall response rate: 75% after 6 months; highest in CAH group (80%)
LNG-IUD vs. Oral Progestins	824 (459 oral, 365 IUD)	EIN	Response: 82% (oral), 95% (LNG-IUD); LNG-IUD more effective, though with study heterogeneity (I² > 50%)

## 8. Progestins in male contraception

**Figure 18.**
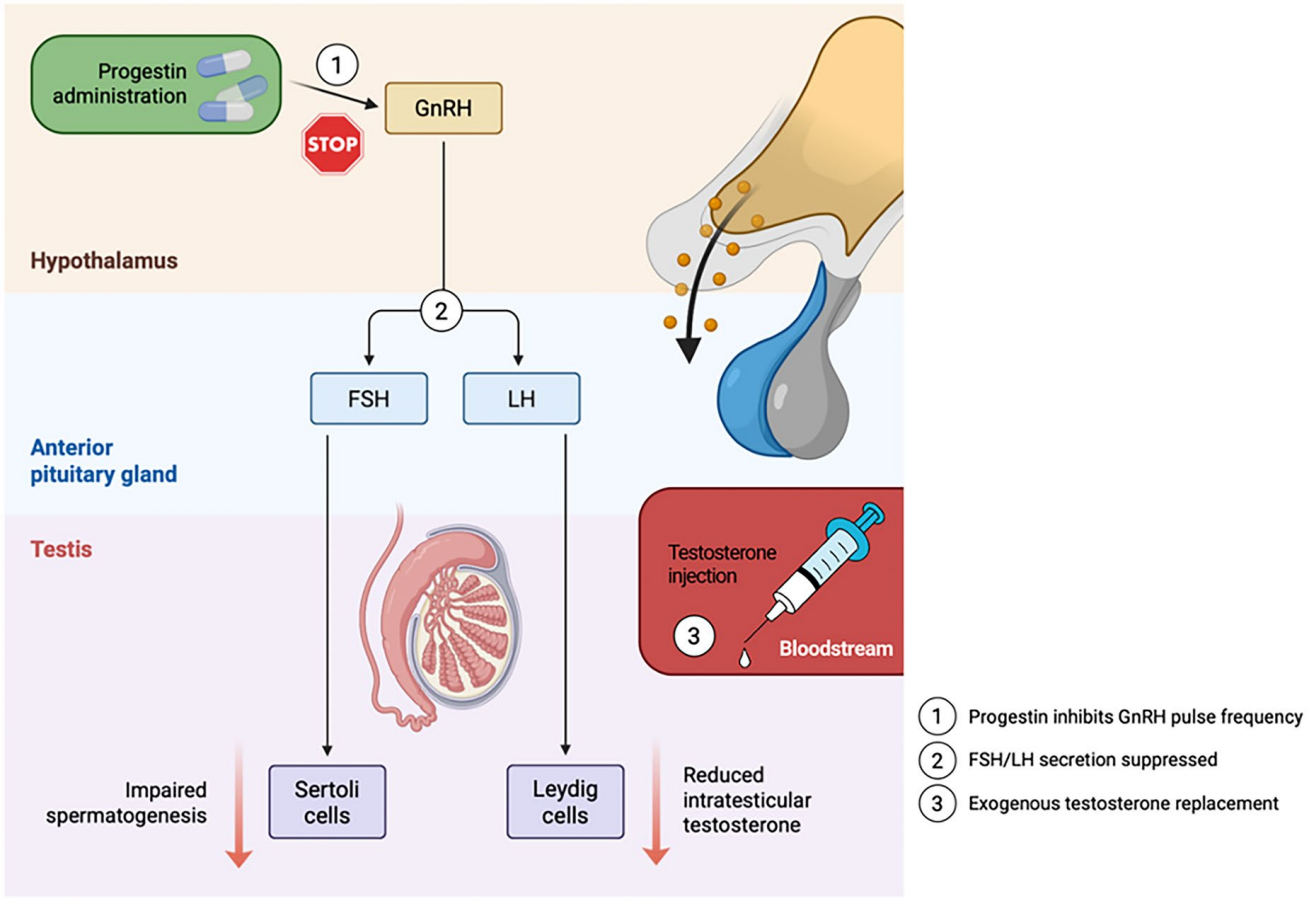
Mechanism of progestin-induced male contraception. Illustrating: (1) Progestin inhibition of GnRH pulsatility, (2) subsequent suppression of FSH/LH secretion, and (3) resultant testicular impairment requiring exogenous testosterone replacement to maintain extragonadal androgenic functions while achieving contraceptive efficacy.

There are known different manners of using progestins as a contraception in women and men. In women, as already mentioned, one of the possible treatment options is to combine progestins with estrogen as a COC. However, in men, a combination of progestins and TE is used [10]. This approach represents a major advance in the development of reversible and effective male contraception [178]. Progestins, acting on the hypothalamus, inhibit the pulsatile release of GnRH, thereby stimulating the pituitary gland to release LH and FSH. Consequently, they disrupt the hypothalamic-pituitary-gonadal axis. Additional exogenous administration of TE results in a stronger suppression of LH and FSH release. FSH is responsible for supporting the Sertoli cells in the testes. Without FSH, spermatogonia in the testes do not develop. LH, in turn, stimulates Leydig cells to produce TE. Without sufficiently high levels of intratesticular TE, spermatogenesis does not proceed properly [179]. Moreover, TE prevents the atrophy of secondary sexual characteristics induced by progestins (progestins lead to a decrease in endogenous TE levels) [180]. Furthermore, progestins in combination with TE lead to apoptosis of the spermatogenic epithelium. After discontinuation of progestins and TE, the contraceptive effect is reversed and spermatogenesis returns to normal [179]. Figure 18 presents the mechanism of progestins’ action in male contraception.

Research has shown that the combination of depot androgens, such as TE, with progestins can effectively suppress spermatogenesis with a relatively low incidence of side effects [63,178]. According to the studies, progestins such as NETA, MPA, DMPA, LNG, NG, ENG, nestrone (NES) and CPA combined with TE have an impact on the suppression of sperm output [181–186]. Table 6 presents known combinations of progestins and TE, as well as preferred administration methods.

**Table 6.** Progestins combined with TE as a male contraception [10,181–185,187].

Combination of androgen and progestin	Administration
TE+MPA	TE+MPA IM (1 per month)
	TE DG (1 per day) + MPA PO (1 per day)
TE+DMPA	TE+DMPA IM (1 per month)
	DTE SC implant + DMPA IM (1 per 3 months)
TE+LNG	TE IM (1 per week) +LNG PO (1 per day)
TE+NETA	TE SC (1 per day) + NETA PO (1 per day)
	TE IM+NETA IM (1 per 8 weeks)
TE+CPA	TE IM (1 per week) +CPA PO (2 per day)
TE+ENG	TE SC (1st day, 24th week) +ENG SC implant (1 per 24 weeks)
TE+NG	TE IM+NG SC implant (no data)
TE+NES	TE+NES DG (1 per day)

TE: testosterone; DTE: depot TE MPA: medroxyprogesterone acetate; DMPA: depot medroxyprogesterone acetate; LNG: levonorgestrel; NETA: norethisterone acetate; CPA: cyprotherone acetate; ENG: etonogestrel; NG: norgestrel; NES: nestrone; IM: intramuscular injection; SC: subcutaneous injection; PO: oral; DG: dermal gel.

Several different forms of drug administration have been studied, including injectable, oral and implantable options. For example, a regimen of 200 mg NETA combined with TE administered every eight weeks, or monthly injections of MPA and TE, has been shown to effectively reduce sperm production [182]. Oral combinations, such as LNG or NET with TE, as well as CPA with TE, have also been shown to be effective [49,188]. Progestogens can also be administered using implants, which provide a sustained and effective method of suppressing spermatogenesis. Non-biodegradable implants containing progestins such as LNG [183] or ENG [189,190] in combination with TE are highly effective in the suppression of sperm production [10].

In addition to their role in male contraception, TE, DMPA, and NETA have been compared for their efficacy in menstrual suppression in transgender males. A significant therapeutic option involves the initial application of TE, followed by the addition of NETA or DMPA. NETA achieves menstrual suppression in a shorter time frame (56 days) compared to DMPA (168 days), making it a preferable choice for adolescents and young adult transgender males [191].

The combination of androgens, such as TE, with progestins effectively induces suppression of spermatogenesis with a relatively low incidence of side effects. However, androgenic effects, including acne, weight gain, sweating, and changes in mood or libido, have been reported. Higher doses of TE may lead to more pronounced side effects, including hepatic impact, reduced SHBG, and reduced HDL levels [192]. Importantly, the suppression of spermatogenesis is reversible. Normal sperm production typically resumes within four to five months of stopping treatment [193,194].

CPA is a synthetic P4 derivative effective with depot androgens for male contraception. Studies have shown that a combination of CPA and TE is more effective [183,192]. The first study involved 15 men aged 22–44. They were randomly divided into the first group receiving CPA (50 mg twice a day, orally) + TE (100 mg/week, im), the second group receiving CPA (25 mg twice a day, orally) + TE (100 mg/week, im) and a third group receiving only TE (100 mg/week, IM). Men in the first and second groups achieved azoospermia, while in the third group, only 3 out of 5 men achieved azoospermia. The time to achieve azoospermia for the first, second and third groups was 6.8 ± 0.5, 8.4 ± 1.0 and 14.0 ± 1.2 weeks. The remaining 2 men did not achieve azoospermia [182]. In turn, the second study involved 10 men aged 19–42. They were randomly divided into the first group receiving CPA (25 mg twice a day, orally) + TE (100 mg/week, im) and the second group receiving CPA (12.5 mg twice a day, orally) + TE (100 mg/week, im). All men from the first group and 3 out of 5 men from the second group achieved azoospermia at 16 weeks. The remaining 2 men never achieved azoospermia [192]. Progestin side effects are inappreciable with the use of a proper dose of TE. Importantly, normal spermatogenesis returns by discontinuation of treatment, consistent with the spermatogenic cycle. The contraceptive effect of CPA resolves after 4–5 months [10,193,195]. Additionally, oral CPA is a component of combined MHT for endometrial protection. No case of EH or EC was detected [8].

## 9. Progestins in new solutions

By the hydrophobic character of the P4 molecule, attempts are constantly being made to improve alternative routes of administration. A study conducted by P. Suryaamporn et al. discusses the development of lipid-based formulations for improved transdermal action with P4. Due to the above, it may be applied in Alzheimer’s disease prevention in the postmenopausal period.

The transdermal system consisted of progesterone-loaded solid-lipid nanoparticles (SLNs) with progesterone-loaded solid-lipid nanoparticles (PG‑SLNs) with the support of the Design of Experiments (DoE) and Artificial Neural Networks (ANN). Ultrasound-assisted emulsification was used to prepare the nanoparticles. The optimal mixture of nanoparticles was determined, comprising 5% stearate, 1.76% medium-chain triglycerides (MCT), 0.30% Pluronic F-127, and 0.5% propylene glycol. Transdermal permeability tests were further conducted using Franz-type diffusion chambers. Three formulations were compared: progesterone suspension, PG-SLNs without enhancer, and PG-SLNs with 2% limonene as a permeability enhancer. The results showed that the formulation with limonene achieved significantly higher diffusion parameters. The lag time for the suspension was 5.33 h, for PG-SLNs without limonene, 2.33 h, and for the formulation with limonene, merely 0.53 h. The permeability coefficient (Kp) for the formulation with limonene reached 19.03 cm/h, which means almost 20 times higher permeation than for the classic progesterone suspension. The developed formulation of SLNs showed high stability, increased homogeneity, significant drug loading and efficiency in transdermal permeation after application of the limonene enhancer. The study confirmed that the combination of the DoE method and ANN could be an effective tool in optimizing advanced drug delivery systems to alleviate neurodegenerative disorders in postmenopausal women [196].

Moreover, N. S. Velasquez et al. decided to develop a vaginal thermosensitive gel with P4. It was decided to use chitosan as the base of the gel, into which an inclusion complex of P4 with methylated β-cyclodextrin was introduced. In this combination, it was achievable to significantly increase the solubility of P4. Particularly if there was a notable concern in vaginal medication forms. In the study, P4 complexes with natural and methylated β-cyclodextrin were prepared by freeze-drying and then introduced into a chitosan thermosensitive gel. The physicochemical properties of the resulting gel-including its macroscopic and microscopic appearance, temperature and time of transition from liquid to gel form (important for vaginal application), as well as the stability of the mixture using porcine vaginal mucosa, were evaluated. The results were compared with the commercially available Crinone^®^, used to treat progesterone deficiency. After 24 h of incubation in artificial vaginal fluid, the new formulation retained its gel structure. Crinone^®^ disintegrated more rapidly.

The conclusions of the study indicate the developed chitosan thermosensitive gel with a progesterone-methylated β-cyclodextrin complex as an effective and more stable alternative to the P4 vaginal formulations currently in use. The advantages of the substance include easy application, adequate release profile and good stability in the physiological environment [197].

## 10. Discussion

In our study, we presented progestins as an extremely broad group of substances used in numerous aspects. A significant number of clinical conditions in which progestins are used have already been thoroughly described in the literature.

Further conditions involve the use of progestins in the oncology treatment of P4-sensitive cancers such as endometrial, renal or breast cancers [11]. However, the use of MA in the therapy of ovarian or prostate cancer is an off-label use, as is anorexia-cachexia syndrome [46]. Additionally, it was noticed that the antiangiogenic effect used in EC to inhibit tumor progression positively affects the treatment of deep endometriosis [174]. Fairly controversial research includes the use of MA in the treatment of hepatocellular carcinoma (HCC). According to some researchers, MA slows down tumor growth (only in PR-positive tumors) and thus improves the survival of palliative patients. Nevertheless, additional studies do not show a significant improvement in the quality of life of patients after using this P4 therapy. MA cannot be clearly defined as a therapeutic agent in the palliative therapy of HCC [129,176]. Moreover, it is suggested that MPA may have a positive influence on the treatment of liver tumors. Whereas, these must be PR-positive metastases of breast cancer because HCC is still a highly controversial subject due to the lack of clear evidence of the effectiveness of the therapy, as well as the lack of understanding of some processes taking place in the liver [176].

The use of progestins in breast cancer remains controversial. There are indications of the possible proliferative and antiproliferative effects of progestins [167]. Due to the above, in our study, we included breast cancer entities both as a probable indication and contraindication to their use [1,11,167]. The suggested effect of progestins has still not been confirmed, which complicates the treatment of patients. Therefore, clinical trials are being conducted on the possibility of using these substances in breast cancer [1,11].

A connection has been noted between the effect of PR agonists and the expression of the estrogen receptor (ER). This is supported by the favorable therapeutic results of patients. In this regard, the PIONEER study focuses on the combination of MA with letrozole. The study included postmenopausal women with newly diagnosed, untreated ER+, HER2-, invasive breast cancer with a size of at least 1 cm. It assumes that patients will be given a low (40 mg) or high (160 mg) dose of MA in combination with letrozole [18]. This is a promising preoperative therapy that reduces tumor mass and offers opportunities for new breast cancer treatment programs.

Another current study suggests increased long-term survival of patients with operable breast cancer through the use of hydroxyprogesterone during surgery. The reports analyzed by the researchers show that performing surgery in the luteal phase of the menstrual cycle significantly improved women’s survival. Therefore, this study aims to create a luteal environment in patients by intramuscular injection of 500 mg of hydroxyprogesterone 5 to 15 days before surgery [19]. The use of hydroxyprogesterone appears to be a favorable prognostic factor for overall survival. P4 may counteract the harmful effects of estrogen during surgery [20]. Concurrently, authors S. Zaami et al. suggest the use of reproductive counselling before initiating breast cancer treatment. In their study, the relationship between diagnosis and the offer of fertility preservation in patients was examined. The study included 51 women aged 31 to 40 diagnosed with breast cancer. According to the collected data, only 21 of the 51 patients (40%) were offered the option of undergoing fertility preservation interventions. This indicates a lack of adequate training among medical personnel to provide reproductive counselling. However, subsequent data reviewed by the authors reveal an upward trend in information about fertility preservation options (an increase of up to 60%) [198].

According to one study, P4 has beneficial effects on ‘thin endometry’ syndrome. P4 therapy increases the thickness of the endometrium and, therefore, increases the implantation capacity. It constitutes a reasonable treatment path for patients with miscarriage. However, depending on the dose, P4 can both raise and lower INF-ץ levels [150]. At low doses, P4 increases IFN-ץ, while at high doses, it decreases IFN-ץ. Interestingly, progestin, which also increases the level of IFN-ץ in high doses, is MPA [152]. Thus, the anti-inflammatory effect of P4 can be modulated, depending on the dose. Nevertheless, P4 is a crucial factor in supporting the luteal phase, especially during IVF, when its endogenous production is impaired. Exogenous hCG supplementation is particularly indicated due to the high pregnancy loss rate (79%) in the mid-luteal phase, when P4 levels are lowest [199]. Therefore, progesterone administered intramuscularly or intravaginally is used as adjunctive therapy [200,201].

In addition to the progestins used in PCOS mentioned in our study, myo-inositol (MI), already well-known in research, may also have a supportive effect. Researchers D. Coldebella et al. report that, in addition to its insulin-sensitizing effects, MI may also participate in pathways responsible for fertility. This improves ovarian stimulation protocols in IVF. Another form, D-Chiro-Inositol (DCI), has anti-inflammatory effects by reducing IL-6 levels and, correspondingly, acts as an antioxidant. This is crucial in infertility associated with endometriosis caused by oxidative stress [202]. Appropriate supplementation, especially based on resveratrol, leads to the production of more mature follicles, as well as a higher number of fertilizations in ICSI [203]. Alpha-lipoic acid (ALA) may exert a supportive effect on MI. Due to its effect on glucose metabolism, ALA may alleviate symptoms associated with PCOS. However, it does not significantly affect hormonal levels. The association between ALA and MI should be further investigated [204]. Interestingly, MI has been shown to affect FSH levels in postmenopausal women. MI causes a decrease in FSH levels and therefore sensitizes cells to insulin [205]. Furthermore, a randomized, double-blind trial was conducted evaluating the combination of MI, boswellia, and betaine in the treatment of mammographic breast density. Highly dense breasts in women increase the risk of developing breast cancer 6-fold compared to low-density breasts. The study included 76 premenopausal women aged 22–51 years with high-density breasts. Patients were randomly assigned to the experimental group or the placebo group. After 6 months, a statistically significantly greater decrease in breast density was observed in the experimental group compared to the placebo group (60% vs. 9%). This indicates the noteworthy clinical importance of substance combination in preventing breast cancer development [206].

## 11. Conclusion

It is possible to select the appropriate therapy due to the broad spectrum of usage forms and the ability to combine them with estrogen or testosterone. Many significant applications are undergoing clinical trials, which promise a positive effect on expanding the prospects of using progestogens. However, some of them may create a legal risk to medics by patients due to their off-label use, which increases the need to propose further guidelines. By using progestogens in the treatment of non-gynecological patients, it is worth considering the development of research on this group of substances also towards the therapy of diseases related to completely different sites in the human body.

## Data Availability

Data sharing is not applicable to this article as no new data were created or analysed in this study.
