# Correction Notice

**DOI:** 10.1080/07853890.2026.2636349

**Published:** 2026-02-26

**Article title:** Progestins - a review of clinical application in gynecology

**Authors:** Dominika Żyła, Dominika Kuźmiuk, Martyna Rynkowska, Małgorzata Satora, Krzysztof Kułak and Rafał Tarkowski

**Journal:** *Annals of Medicine*

**Bibliometrics:** Volume 57, Number 01, 2570794

**DOI:** https://doi.org/10.1080/07853890.2025.2570794

When the above article was first published online, two references were inadvertently merged into a single reference entry. This has now been corrected, and the article has been republished. The details of the correction are provided below.

**Reference correction**

Reference 174 was previously presented as a combined entry. It has now been split into two separate references, and all subsequent references have been renumbered accordingly.

Previously published as:

174. Powell SG, Sharma P, Masterson S, Wyatt J, Arshad I, Ahmed S, Lash G, Cross M, Hapangama DK. Vascularisation in deep endometriosis: a systematic review with narrative outcomes. Cells. 2023 12(9):1318.; Yeh YT, Chang CW, Wei RJ, Wang SN. Progesterone and related compounds in hepatocellular carcinoma: basic and clinical aspects. Biomed Res Int. 2013;2013:290575. doi:10.1155/2013/290575. doi:10.3390/cells12091318.

Corrected to:

174. Powell SG, Sharma P, Masterson S, Wyatt J, Arshad I, Ahmed S, Lash G, Cross M, Hapangama DK. Vascularisation in deep endometriosis: a systematic review with narrative outcomes. Cells. 2023;12(9):1318. doi:10.3390/cells12091318.

175. Yeh YT, Chang CW, Wei RJ, Wang SN. Progesterone and related compounds in hepatocellular carcinoma: basic and clinical aspects. Biomed Res Int. 2013;2013:290575. doi:10.1155/2013/290575.

**Citation correction**

In the following sentence, the citation range has been updated to reflect the renumbering:

Previously published as:

“They may be an effective therapeutic option for patients with advanced, recurrent endometrial cancer (with low toxicity and positive P4 receptor), as well as in the treatment of deep endometriosis [168–174].”

Corrected to:

“They may be an effective therapeutic option for patients with advanced, recurrent endometrial cancer (with low toxicity and positive P4 receptor), as well as in the treatment of deep endometriosis [168–175].”

All subsequent citations have been renumbered accordingly.

